# Supplementary material for: Differential Coupling of Adult-Born Granule Cells to Parvalbumin and Somatostatin Interneurons
Source: Cell Rep. Author manuscript; Available in PMC 2020 Feb 11. (PMC7011182; doi:10.1016/j.celrep.2019.12.005)
Supplement: 1 [file NIHMS1548573-supplement-1.pdf]

Cell Reports, Volume *30*

## Supplemental Information

# Differential Coupling of Adult-Born Granule Cells to Parvalbumin and Somatostatin Interneurons

**Ayelén I. Groisman, Sung M. Yang, and Alejandro F. Schinder**

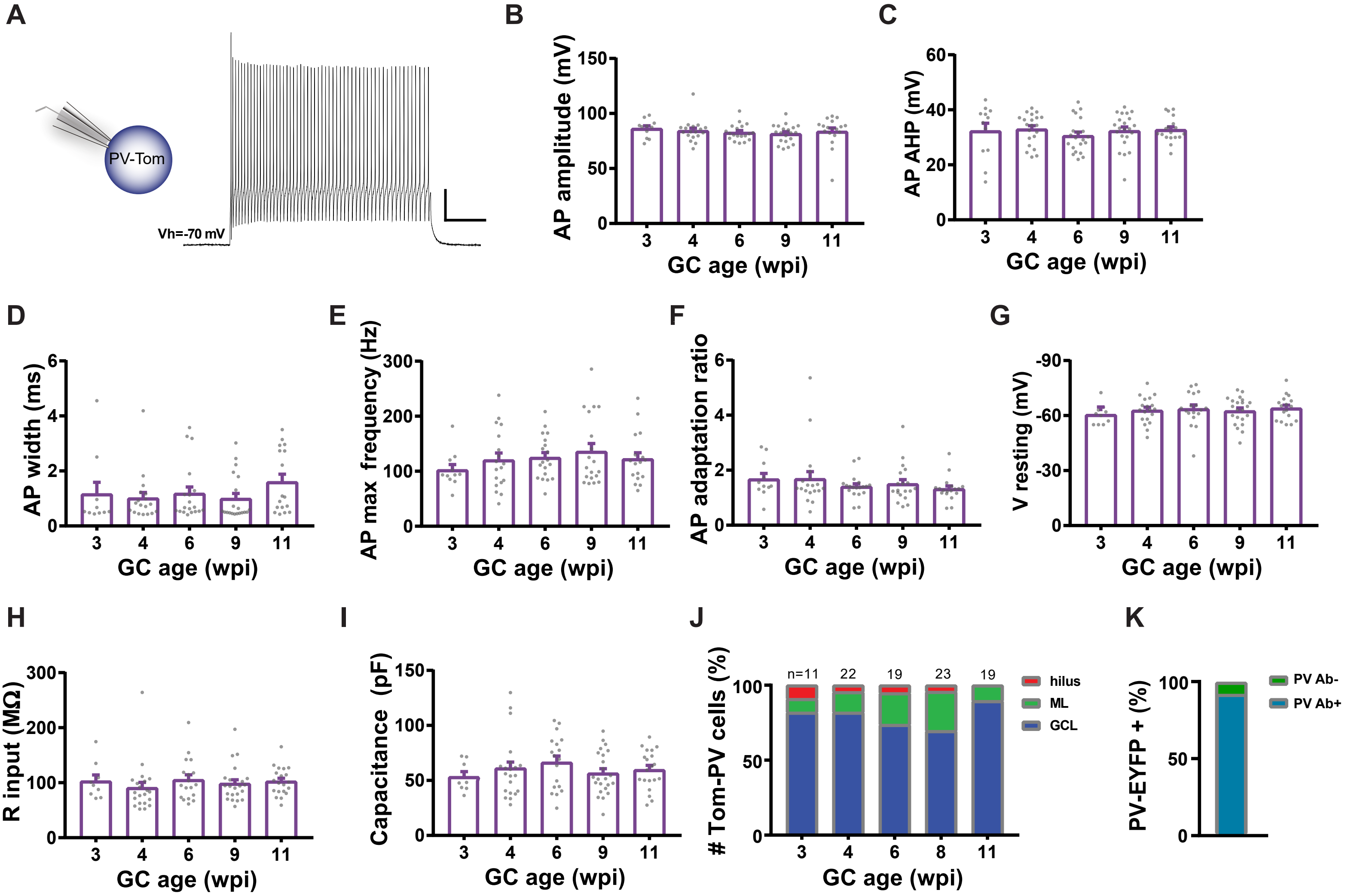
A
PV-Tom
Vh=-70 mV
B
AP amplitude (mV)
GC age (wpi)
C
AP AHP (mV)
GC age (wpi)
D
AP width (ms)
GC age (wpi)
E
AP max frequency (Hz)
GC age (wpi)
F
AP adaptation ratio
GC age (wpi)
G
V resting (mV)
GC age (wpi)
H
R input (MΩ)
GC age (wpi)
I
Capacitance (pF)
GC age (wpi)
J
# Tom-PV cells (%)
n=11
22
19
23
19
hilus
ML
GCL
GC age (wpi)
K
PV-EYFP + (%)
PV Ab-
PV Ab+

**Figure S1. Characterization of dentate gyrus PV-INs. Related to Fig. 1.** (A) (left) Experimental scheme showing whole-cell recordings on Tom-PVs. (Right) PV-IN recording displaying high frequency firing in response to 600 pA-step. Scale bar: 100ms, 20mV. Spiking profile was assessed using the following measures: action potential (AP) amplitude (B), after hyperpolarization amplitude (C), AP width (D), firing maximum frequency (E), and AP adaptation ratio (F). Membrane passive properties are described by resting membrane potential (G), input resistance (H) and membrane capacitance (I). Sample sizes were 11-23 cells in 5-9 animals. Statistical comparisons were done using Kruskal-Wallis test. (J) Recorded Tom-PVs were characterized by their location in the dentate gyrus, divided into three main areas: hilus, molecular layer (ML) and granular cell layer (GCL). The spatial distribution of recorded Tom-PVs is not significantly different among groups. The total number of cells are shown on top of each column. Statistical comparisons were done using Chi-square test ($p$ = 0.819). (K) Proportion of ChR2-expressing PV-INs (PV-EYFP$^{+}$) labeled with an antibody for PV (PV Ab). N = 90 cells from 3 mice. No PV-EYFP cells colocalized with an antibody for SST-Ab (n = 142 cells).

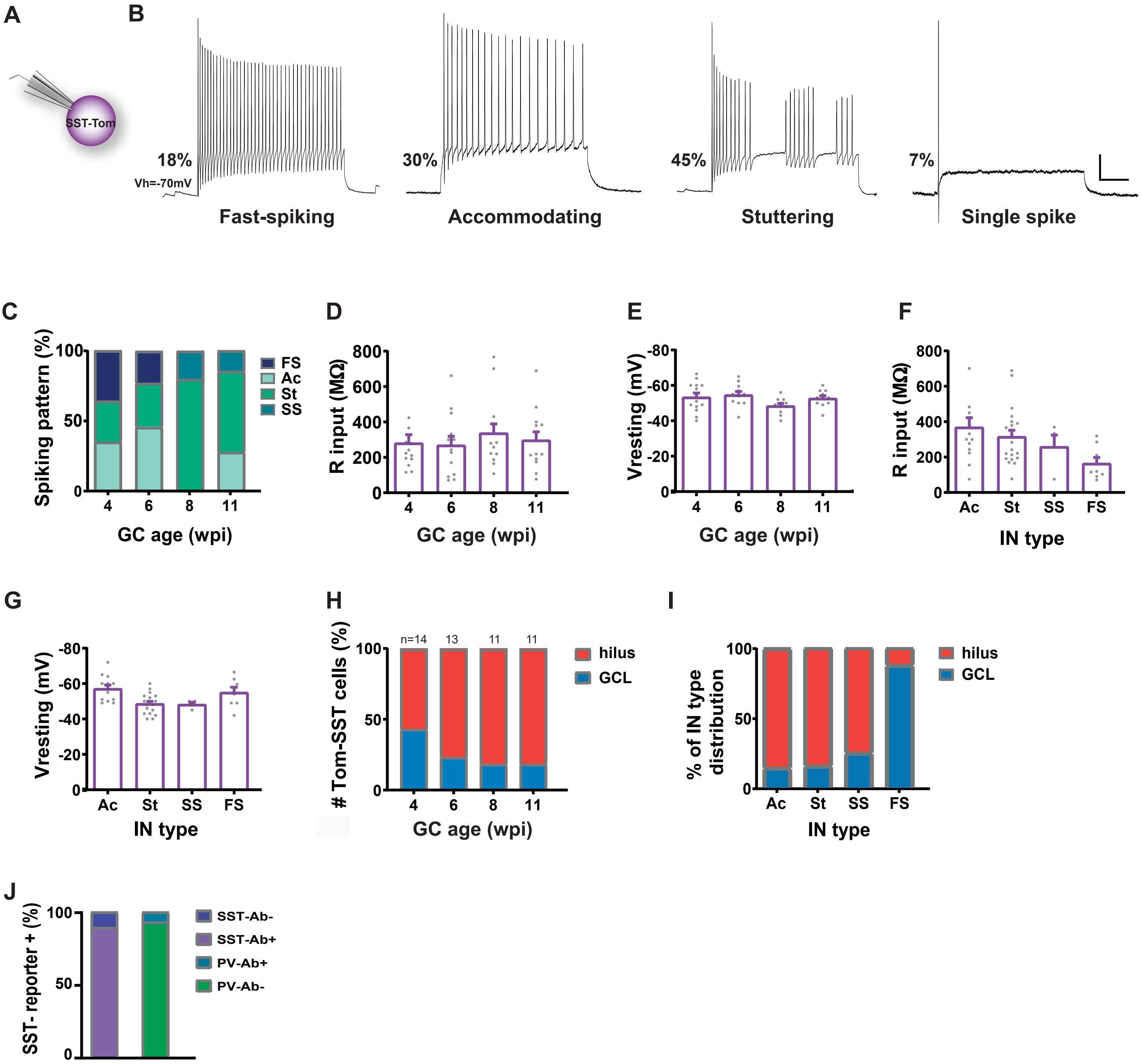
A
SST-Tom
B
18%
Vh=-70mV
Fast-spiking
30%
Accommodating
45%
Stuttering
7%
Single spike
C
Spiking pattern (%)
100
50
0
4
6
8
11
GC age (wpi)
FS
Ac
St
SS
D
R input (MΩ)
800
600
400
200
0
4
6
8
11
GC age (wpi)
E
Vresting (mV)
-80
-60
-40
-20
0
4
6
8
11
GC age (wpi)
F
R input (MΩ)
800
600
400
200
0
Ac
St
SS
FS
IN type
G
Vresting (mV)
-80
-60
-40
-20
0
Ac
St
SS
FS
IN type
H
# Tom-SST cells (%)
n=14
13
11
11
100
50
0
4
6
8
11
GC age (wpi)
hilus
GCL
I
% of IN type distribution
100
50
0
Ac
St
SS
FS
IN type
hilus
GCL
J
SST- reporter + (%)
100
50
0
SST-Ab-
SST-Ab+
PV-Ab+
PV-Ab-

**Figure S2. Characterization of dentate gyrus SST-INs. Related to Fig. 1.** (A) Experimental scheme for whole-cell recordings on Tom-SSTs. (B) SST-IN recording showing repetitive firing in response to 510 pA step. We observed four different spiking profiles: fast spiking (FS), accommodating (Ac), stuttering (St) and single-spike (SS). Proportions (%) corresponding to each spiking profile are indicated. Scale bar: 100ms, 20mV. (C) Percentage of SST-INs spiking profiles recorded at different GC stages. Membrane passive properties are described by input resistance (D) and membrane resting potential (E). Passive properties were then classified for each spiking profile: input resistance (F) and membrane resting potential (G). (H) Distribution of recorded SST-INs within the dentate gyrus for the indicated ages, corresponding to Fig. 1. Total number of cells are shown on top of each column. (I) The spatial distribution for INs presenting each spiking profile is similar except for FS, which is mainly located in the GCL. (J) Proportion of ChR2-expressing SST-INs (SST-EYFP$^{+}$) labeled with antibodies for SST (N = 83 cells, 2 mice) or PV (N = 103 cells, 2 mice).

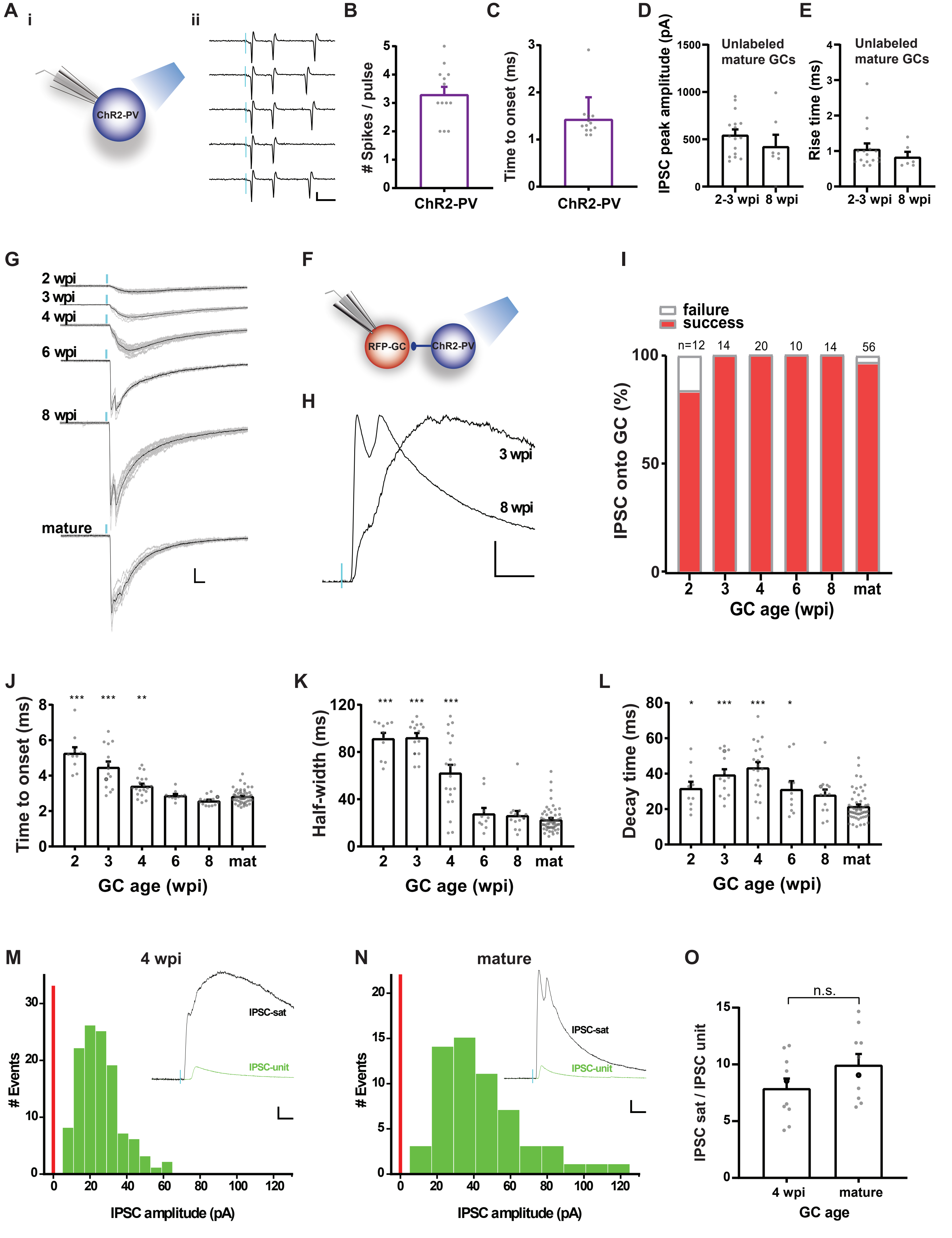

A
i
ii
ChR2-PV
B
# Spikes / pulse
ChR2-PV
C
Time to onset (ms)
ChR2-PV
D
IPSC peak amplitude (pA)
Unlabeled mature GCs
2-3 wpi
8 wpi
E
Rise time (ms)
Unlabeled mature GCs
2-3 wpi
8 wpi
G
2 wpi
3 wpi
4 wpi
6 wpi
8 wpi
mature
F
RFP-GC
ChR2-PV
H
3 wpi
8 wpi
I
failure
success
n=12
14
20
10
14
56
IPSC onto GC (%)
GC age (wpi)
2
3
4
6
8
mat
J
Time to onset (ms)
GC age (wpi)
K
Half-width (ms)
GC age (wpi)
L
Decay time (ms)
GC age (wpi)
M
4 wpi
IPSC-sat
IPSC-unit
# Events
IPSC amplitude (pA)
N
mature
IPSC-sat
IPSC-unit
# Events
IPSC amplitude (pA)
O
n.s.
IPSC sat / IPSC unit
4 wpi
mature
GC age

**Figure S3. In-depth characterization of IPSCs evoked by ChR2-PV activation. Related to Fig. 1.** (A) PV-INs spiking elicited by optogenetics. (i) Experimental scheme shows PV-INs recording after brief laser pulses. (ii) Cell attached recording showing PV-INs reliable spiking after single laser pulse stimulation (0.2 ms, 0.07 Hz, blue marks). Scale bar, 20 pA, 5 ms. (B) Total number of spikes per single pulse when applying saturating intensity of laser stimulation. All measured ChR2-PVs were responsive to single laser pulses. N = 13 cells / 9 mice. (C) Time to onset of the first spike. (D, E) Properties of IPSCs recorded from unlabeled mature GCs present in the same slices as 2-3 wpi or 8 wpi GCs. (F) Experimental scheme for RFP-GC recording after PV-IN stimulation. (G) IPSCs elicited by laser pulses (0.2 ms) delivered at low frequency (0.07 Hz), recorded from GCs at the indicated ages. Traces depict individual sweeps (gray) and their average (black). Scale bars, 100 pA, 10 ms. (H) Normalized traces highlighting the differences in kinetics for responses recorded from GCs at 3 and 8 wpi. Scale bar, 10 ms, 0.2 au. (I) Percentage of adult-born GCs displaying a postsynaptic response after activation of PV-INs. Total amount of GCs recorded are shown on top of each column. (J-L) Properties of IPSCs elicited by PV-INs onto GCs at the indicated ages. (M, N) Representative histogram of IPSC amplitude evoked by minimal stimulation for 4 wpi (M) and mature GCs (N). Insets show normalized average traces in response to minimal (IPSC-unit) and maximal (IPSC-sat) stimulation. Scale bars, 10 ms, 0.1 au. (O) The number of IPSC-units contained in IPSC-sat are not different in immature vs. mature GCs. Black circles correspond to the examples shown in M, N. n = 10 (4 wpi) and 10 (mature) cells. Statistical comparison was done using two-tailed *t*-test (p=0.128). Comparisons for (J) to (L) were done using one-way ANOVA followed by *post hoc* Bonferroni's test for multiple comparisons against the mature condition; (*), (**) and (***) denote $p<0.05$, $p<0.01$ and $p<0.001$.

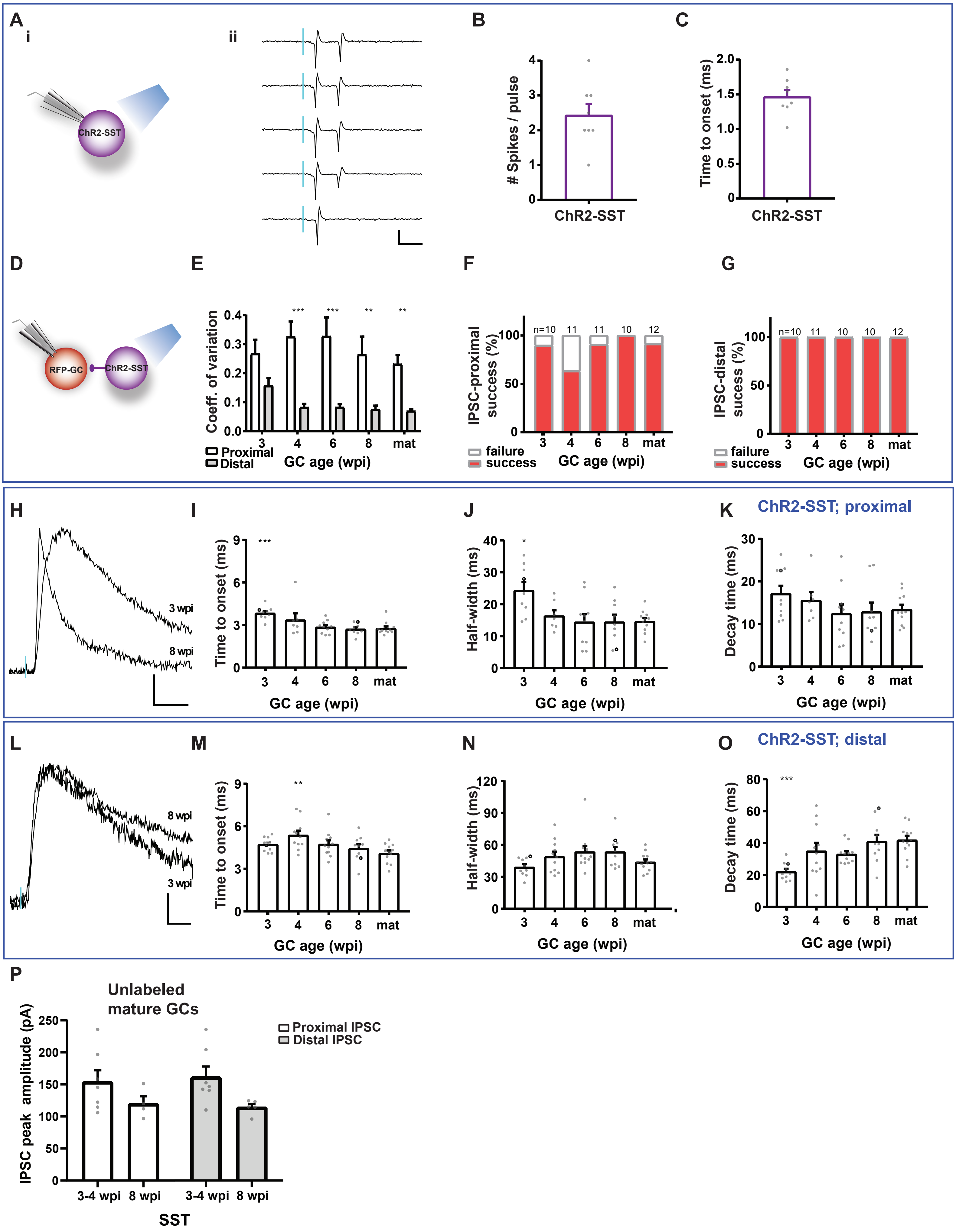
A
i
ii
ChR2-SST
B
# Spikes / pulse
ChR2-SST
C
Time to onset (ms)
ChR2-SST
D
RFP-GC
ChR2-SST
E
Coeff. of variation
*** *** ** **
Proximal
Distal
GC age (wpi)
F
IPSC-proximal success (%)
n=10 11 11 10 12
failure
success
GC age (wpi)
G
IPSC-distal sucess (%)
n=10 11 10 10 12
failure
success
GC age (wpi)
H
3 wpi
8 wpi
I
Time to onset (ms)
***
GC age (wpi)
J
Half-width (ms)
*
GC age (wpi)
K
ChR2-SST; proximal
Decay time (ms)
GC age (wpi)
L
8 wpi
3 wpi
M
Time to onset (ms)
**
GC age (wpi)
N
Half-width (ms)
GC age (wpi)
O
ChR2-SST; distal
Decay time (ms)
***
GC age (wpi)
P
Unlabeled mature GCs
IPSC peak amplitude (pA)
Proximal IPSC
Distal IPSC
3-4 wpi 8 wpi 3-4 wpi 8 wpi
SST

**Figure S4. In-depth characterization of IPSCs evoked by ChR2-SST activation. Related to Fig. 1 and 2.** (A) SST-INs spiking elicited by optogenetic. (i) Experimental scheme shows SST-INs recording after laser stimulation. (ii) Representative cell attached recordings showing SST spiking after single laser-pulse stimulation (0.2 ms, 0.07 Hz, blue marks). Scale bar, 20 pA, 5 ms. (B) Total number of spikes per single pulse. All measured ChR2-SSTs were responsive to a single-pulse laser stimulation. N = 8 cells / 5 mice. (C) Time to onset of the first SST-IN spike. (D) Simplified experimental schematic shows RFP-GCs recording when ChR2-SSTs are stimulated. (E) Coefficient of variation for proximal and distal IPSC amplitudes recorded in adult-born GCs. Statistical comparisons were done using two-way ANOVA followed by *post hoc* Bonferroni's test for multiple comparisons. (F, G) Percentage of adult-born GCs presenting response to activation of SST-INs, for both proximal (F) and distal (G) IPSCs. Total number of recorded GCs are shown on top of each column. Statistical comparisons were done using Fisher's exact test. (H-K) Proximal IPSC kinetics. (H) Normalized traces highlighting the differences in kinetics for responses evoked in adult-born GCs at 3 and 8 wpi. Scale bars, 10 ms, 0.2 norm. We measured time to onset (I), half-width (J) and decay time (K). (L-O) Distal IPSC kinetic, corresponding to *H-K*. Statistical comparisons were done using Kruskal-Wallis test followed by Dunn's multiple comparisons against mature condition (I-K, N-O) and one-way ANOVA followed by post-hoc Bonferroni's test (M). (P) Amplitude of IPSCs recorded from unlabeled mature GCs present in the same slices as 3-4 wpi or 8 wpi GCs. For all statistical tests, (*), (**) and (***) denote $p$<0.05, p<0.01 and $p$<0.001.

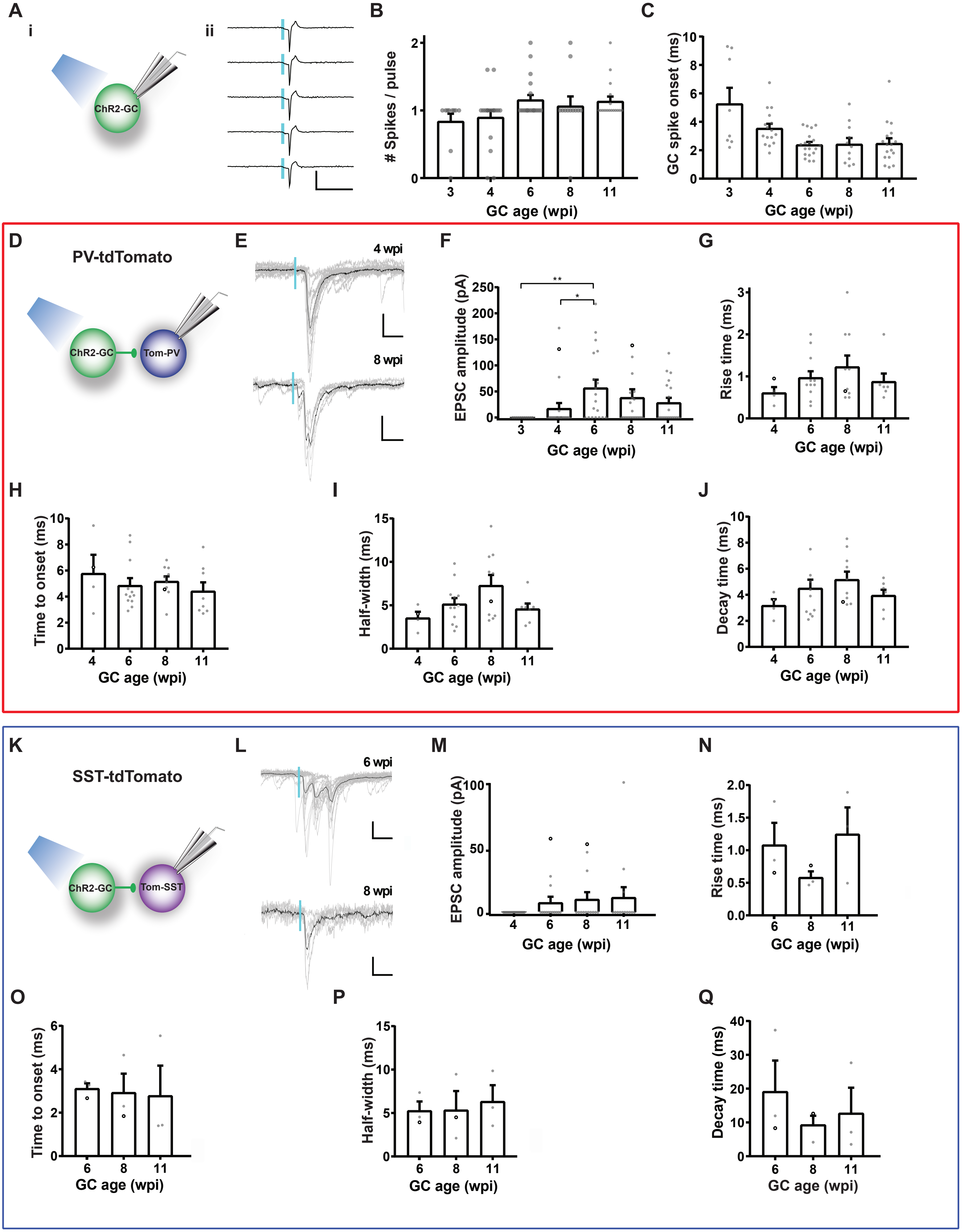
A
i
ii
ChR2-GC
B
# Spikes / pulse
GC age (wpi)
C
GC spike onset (ms)
GC age (wpi)
D
PV-tdTomato
ChR2-GC
Tom-PV
E
4 wpi
8 wpi
F
EPSC amplitude (pA)
GC age (wpi)
G
Rise time (ms)
GC age (wpi)
H
Time to onset (ms)
GC age (wpi)
I
Half-width (ms)
GC age (wpi)
J
Decay time (ms)
GC age (wpi)
K
SST-tdTomato
ChR2-GC
Tom-SST
L
6 wpi
8 wpi
M
EPSC amplitude (pA)
GC age (wpi)
N
Rise time (ms)
GC age (wpi)
O
Time to onset (ms)
GC age (wpi)
P
Half-width (ms)
GC age (wpi)
Q
Decay time (ms)
GC age (wpi)

**Figure S5. In-depth characterization of EPSCs evoked onto PV- and SST-INs by ChR2-GC activation. Related to Fig. 5.** (A) ChR2-GCs spiking elicited by optogenetics. (i) Experimental scheme of GCs recording after optogenetic stimulation. (ii) Representative cell-attached recordings in ChR2-GCs show reliable spiking evoked by brief laser pulse (1ms, 0.07Hz, blue marks). Representative data from 4 wpi GCs. Scale bar, 10 ms, 50 pA. (B) Total number of spikes per single pulse at each GC stage. Gray dots correspond to single neurons. Sample sizes were 10-19 neurons in 5-9 mice. (C) Time to onset of the first spike elicited on ChR2-GCs by single laser-pulse stimulation. Activation of adult-born GCs elicits EPSCs onto PV-INs (D-J) and SST-INs (K-Q). (D) Experimental scheme depicting laser-activated GCs and PV-IN recording. (E) Recordings of PV-INs EPSCs elicited by laser-pulse stimulation of adult-born GCs at 4 and 8 wpi. Traces depict all sweeps in the experiment (gray) and their average (black). Scale bar, 10 ms, 50 pA. EPSC peak amplitude (F), rise time (G), time to onset (H), half-width (I) and decay time (J) are presented. Sample sizes were 11-23 neurons in 5-9 mice. (K) Experimental scheme for SST-INs recording. (L) Recordings of SST-INs EPSCs elicited by GCs activation at 6 and 8 wpi. Scale bar, 10 ms, 50 pA (top), 25 pA (bottom). (M-Q) Corresponding to *F-J* for SST-INs recordings. Sample sizes were 12-17 neurons in 2-7 mice. Hollow symbols correspond to example traces. Statistical comparisons were done using Kruskal-Wallis test followed by *post hoc* Dunn's multiple comparisons. $p<0.05$ (*) and $p<0.01$ (**).
